# Rhythmic cognition in humans and animals: distinguishing meter and pulse perception

**DOI:** 10.3389/fnsys.2013.00068

**Published:** 2013-10-31

**Authors:** W. Tecumseh Fitch

**Affiliations:** Department of Cognitive Biology, School of Life Sciences, University of ViennaVienna, Austria

**Keywords:** rhythm, meter, music cognition, cognitive biology, comparative cognition, hierarchy

## Abstract

This paper outlines a cognitive and comparative perspective on human rhythmic cognition that emphasizes a key distinction between pulse perception and meter perception. Pulse perception involves the extraction of a regular pulse or “tactus” from a stream of events. Meter perception involves grouping of events into hierarchical trees with differing levels of “strength”, or perceptual prominence. I argue that metrically-structured rhythms are required to either perform or move appropriately to music (e.g., to dance). Rhythms, from this metrical perspective, constitute “trees in time.” Rhythmic syntax represents a neglected form of musical syntax, and warrants more thorough neuroscientific investigation. The recent literature on animal entrainment clearly demonstrates the capacity to extract the pulse from rhythmic music, and to entrain periodic movements to this pulse, in several parrot species and a California sea lion, and a more limited ability to do so in one chimpanzee. However, the ability of these or other species to infer hierarchical rhythmic trees remains, for the most part, unexplored (with some apparent negative results from macaques). The results from this animal comparative research, combined with new methods to explore rhythmic cognition neurally, provide exciting new routes for understanding not just rhythmic cognition, but hierarchical cognition more generally, from a biological and neural perspective.

## Introduction

The cognitive biology of music has witnessed major advances in the last two decades, due mainly to an explosion of neuroimaging, developmental, and comparative research. But while certain pockets, such as the study of harmonic “syntax,” have received considerable attention, others remain relatively neglected. In this paper I focus on one of these neglected topics: the biological and computational basis of rhythmic cognition, particularly metrical structure. I suggest that the production and perception of rhythm provides a number of important parallels with linguistic phonology and syntax, exemplifying some of the key components of hierarchical processing in a particularly simple and clear manner. I will review recent comparative data indicating that crucial aspects of rhythmic cognition are present in non-human animals. These new findings open the door to investigations of the biological roots and neural implementation of rhythmic processing, aspects which have proven difficult to explore for distinctively human aspects of cognition like language. I end with some suggestions about possible neural substrates of rhythmic and metrical processing.

This paper has three main parts. I first lay out an explicit model of rhythm and rhythmic processing, clarifying the parallels between rhythmic and linguistic syntax as sub-types of hierarchical processing. In particular, structures in both domains are naturally represented as trees, in which every sub-tree has a “head” or dominating node. The notion of headedness plays an important role in both language and music, but in rhythmic (metrical) trees heads are implemented in a particularly clear and simple fashion. Second, I concisely review the fast-growing literature on animal rhythmic capabilities, which suggests that important components of human rhythmic cognition (including beat-finding and synchronization) are shared with various non-human species. I end with some brief neuroanatomical observations, emphasizing differences between chimpanzee and human brains that may point toward the locus of hierarchical processing in the mammalian brain. I conclude that the tendency in much recent literature to equate “musical syntax” with harmony (e.g., Koelsch et al., 2000; Maess et al., 2001; Patel, 2003, 2008) is overly narrow, and risks overlooking the rich and important role rhythmic cognition plays in music, and should play in investigations of the neuroscience of music (cf. Fitch, 2006, 2012; Patel, 2006; Honing, 2009).

There is an extensive empirical literature on various aspects of human rhythmic cognition, including both perception and production, that I will not attempt to review here (Fraisse, 1978; Povel and Essens, 1985; Jones and Boltz, 1989; Desain and Windsor, 2000; Drake et al., 2000; Repp, 2005; McAuley et al., 2006; Honing, 2009). I know of no comprehensive overview of this entire literature, but (Honing, 2009) provides a brief and very accessible introduction to the viewpoint on rhythmic cognition adopted here. Repp (2005); (Repp and Su, 2013) thoroughly reviews the human tapping literature, and (Fraisse, 1978; Clarke, 1999) survey the older literature on rhythmic cognition.

## Rhythmic processing: a form of hierarchical cognition

“Rhythm” and “meter” are dangerously polysemic terms (cf. Sachs, 1953), with interpretations ranging from the very simple (e.g., “the rhythm of the seasons,” which connotes nothing more than simple periodicity) to quite complex (the notion of rhythm and meter employed in Western musical theory). The term “rhythms of the brain” (e.g., Buzsaki, 2006) lies somewhere between, connoting not just periodicity but also a capacity for synchronization. The ambiguity of this term leads to different researchers and different disciplines having quite different ideas about what it means to be “rhythmic” and thus, what, exactly, “rhythmic cognition” entails.

**Figure 1:**
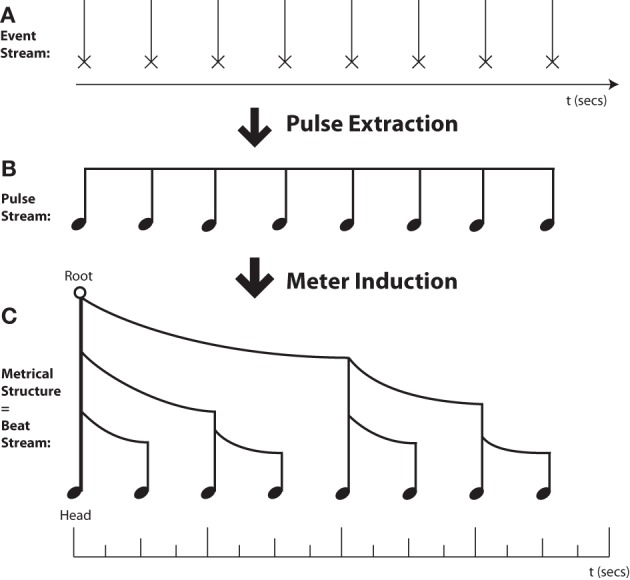
**Musical Rhythms are Trees in Time. (A)** Rhythmic processing starts with an unprocessed sequence of auditory events. **(B) “Pulse Extraction”**—Cognitive processes lead to conversion of a periodic event sequence to an (unaccented) isochronic pulse stream. **(C) “Meter Induction”**—Cognitive processes lead to conversion of an event stream or unaccented pulse stream to a hierarchically-grouped metrical tree structure. The height of each metrical stem indicates the relative prominence of that event, while the curved beams indicate grouping structure. Note that while pulse extraction and meter induction are typically combined in music, these are separate cognitive processes. A pulse can be extracted without any definite meter assignment (e.g., in metrically ambiguous sequences), and meter can be inferred in the absence of isochrony (e.g., in spoken language and poetry).

Here I adopt the complex notion of rhythm used by many music theorists, which has two major components (see Figure 1). First, rhythmic cognition typically involves extracting a “**pulse**” or “tactus” at a particular rate (the tempo) that serves as a basis for organizing and structuring incoming sonic events. Whenever one can dance to or tap one's foot along with a piece of music at the appropriate tempo, one has found this pulse. Even this apparently simple “pulse extraction” ability conceals considerable complexity (cf. Povel, 1984; Merker et al., 2009; Fitch, 2012), and was believed until recently to be uniquely human among mammals (Williams, 1967; but cf. Merker, 2000).

Beyond pulse extraction, musical rhythm further entails a hierarchical grouping of sonic events into well-defined **metrical patterns**, of strongly or weakly accented events (Longuet-Higgins, 1979; Temperley, 2001; Honing, 2012), with clear parallels in language (“metrical phonology,” where the events are syllables) and perhaps other domains such as vision (Jackendoff and Lerdahl, 2006). This overall notion is termed “meter” and is concisely indicated by the time signature in Western musical notation.

Both pulse and meter are *cognitive constructs*, inferred by a listener, and are not explicitly present in the raw acoustic signal. Thus, rhythm, like pitch, is strictly speaking a mental construct which is related but not identical to aspects of the signal (e.g., its frequency). In *finding the pulse* of an incoming series of events, the listener must infer a pulse frequency (which for complex rhythms is not the same as the frequency of notes or other events). *Inferring the meter* entails choosing a particular location in the stream as the downbeat, which then serves as the dominant or “head” node around which to build a hierarchical structure. Assigning a meter to the rhythm also entails inferring some recurring grouping structure that will be applied to incoming events. For example, waltz time (3:4) involves grouping events into groups corresponding to three quarter notes (crotchets), where the first quarter serves as the downbeat. To dance properly to the waltz, listeners must feel this grouping and recognize the downbeat (at least implicitly). In contrast, most rock music uses 4:4 time, which involves groups of four quarter notes, and is danced accordingly. Although terms like “meter” and “time signature” are foreign to many non-musicians, the underlying concepts are (at least implicitly) available to anyone who can dance appropriately in waltz, jitterbug, macarena, or gangnam styles.

I will now attempt to clarify these concepts more formally, emphasizing the relationship between rhythmic and linguistic syntax (encompassing both phonology and phrasal syntax). These basic notions were already clearly laid out by Longuet-Higgins and colleagues in the 1970's (Longuet-Higgins, 1976, 1979; Steedman, 1977), see also (Desain and Honing, 1999), but have unfortunately received less attention than they deserve. Importantly, although the model of metrical structure adopted here stems from theoretical and computational work, its basic validity has also been validated empirically in what little experimental work has been done on syncopation and metrical cognition. For example, Fitch and Rosenfeld found strong correlations between both error rate and frequency of “resetting” with syncopation indices of rhythms, calculated using Longuet-Higgins and Lee's theory-derived model (Fitch and Rosenfeld, 2007). While more empirical work is clearly needed, this suggests that the theoretical models are grounded in psychological and neural reality.

### Pulse perception and metrical structure attribution: a key distinction

I start by clearly distinguishing the processes of pulse finding and meter attribution, since it is possible to do either without doing the other. Henceforth I will adopt the following bi-partite subdivision of rhythmic cognition (Figure 1):

#### Pulse-finding and entrainment

An important characteristic of musical rhythm, not typically present in language, is **isochronicity**: the presence of an even, periodic pulse. While this might be a simple, repeated click (as in a metronome), more typically this pulse is concealed at the musical surface, because not all pulses are played and there are typically additional sonic events that are not on the pulse (cf. Large, 2000; Merker et al., 2009).

When listeners extract a pulse from the acoustic surface, and adjust their own behavior to it (whether their own acoustic output, in ensemble playing, or their movements, as in dance) this is called **entrainment**. An important special case of entrainment occurs when the movements and pulses happen simultaneously: this is termed **synchronization** (which is just entrainment at 0° phase). As we will see below, a capacity for both entrainment and synchronization has been clearly documented in non-human animals.

Although it is very common in music from around the world, an isochronic pulse is not always present. A simple behavioral assay for the presence of a pulse in a musical style involves asking someone experienced in the style to clap along with the beat (Arom, 1991): the “clapping test.” If experienced performers proclaim this task to be inappropriate (e.g., for some forms of chant, or solo instrumental performance), the music presumably lacks an isochronous pulse (sometimes termed “unmeasured”). Note also that in most forms of music the pulse is only **quasi-periodic**: it does not follow a perfectly regular time. In some styles the pulse can be slowed down or sped up considerably for musical effect (e.g., *rubato* and *accelerando* in classical music).

#### Attribution of metrical structure

Once a listener has “found” the pulse, musical rhythmic cognition typically involves a second crucial step: the assignment of a metrical structure to individual pulses, which involves systematic attribution, to each pulse, of a particular level of **perceptual prominence**. That is, we hear some pulses as “more important” than others, *even if they are perceptually identical*. Thus, we can assign a metrical structure of {**strong**, weak, **strong**, weak …} even to the series of identical clicks from a metronome. Crucially, it is often assumed in both music theory and linguistics (metrical phonology, see below) that this metrical structure is **hierarchical**: it is not simply a flat serial pattern, but involves the unconscious creation of a tree structure (Figure 1C). The basic notion of hierarchical metrical structure is shared by music and language (Liberman and Prince, 1977; Fabb and Halle, 2012; Vaux and Myler, 2012) and their similarity is particularly evident in poetry and song lyrics, which both occupy an intermediate ground between music and “normal” language. Unlike pulse-finding, an ability clearly shared by some non-human animals (see below), it remains unclear whether non-human species assign hierarchical structure to a stream of pulses.

Why do many theorists argue that metrical structure is hierarchical? Because the prominence of an event is determined *not* by its serial location (e.g., by rules like “note number 4 in a measure is strong”), but rather by its place in the overall hierarchy of that measure. Thus, a note at a particular branch location of the tree has the same prominence regardless of its serial numbering, and regardless of whether it is preceded by rests, a quarter note, or a string of sixteenth notes. Furthermore, an event's prominence differs depending on the meter assigned by the listener (e.g., 3:4 vs. 4:4 vs. 6:8). These central features of meter are natural consequences of a hierarchical, rather than serial, interpretation of an incoming stream of events (that is, notes or rests in music, and syllables in speech).

Although it is common to find the term “beat” used as equivalent to “pulse,” and the term “rhythm” used for simple pulse-extraction, I think this usage fails to clearly differentiate between the two components distinguished above. It is indeed true that, for human listeners, these two processes are often tightly linked: in rhythmic music, both pulse and beat attribution typically occur simultaneously. Nonetheless, in music one can hear a pulse but be uncertain about the meter, either in terms of grouping (“is this 3:4 or 4:4?”) or about where the downbeat is (“where is the one?”). In speech, particularly in spoken poetry, meter often plays a central role, but isochronicity (and therefore, a regular pulse) is not typically present. Furthermore, from a computational point of view these are two very different processes, one involving periodicity detection (e.g., using spectral decomposition and tempo estimation) and the other involving hierarchical structure building (Longuet-Higgins, 1979; Tomic and Janata, 2008). Finally, while pulse perception is clearly present in non-human species, we still do not know about meter assignment. Below, I will use the term “musical rhythm” to connote *both* pulse extraction and metrical structuring, reserving the term “beat” (as opposed to pulse) for situations in which a stream of musical pulses (or in speech a stream of syllables) have had a metrical structure assigned to them by a listener.

Crucially, just as the periodicity of the pulse may be varied for musical effect (rubato), a composer or performer may insert deviations from the overall metrical structure into a musical performance. Such metrical anomalies are generally termed **syncopations**. Once a metrical context is established, involving alternations of strong and weak events, musicians can “play” within this context by deviating from rhythmic expectations. This type of “expectancy violation” can add an element of surprise, energy or excitement to music, but if taken too far can lead to a breakdown of the inferred metrical structure. This forces listeners to “reset” their metrical expectations to a new, less syncopated metrical interpretation (Longuet-Higgins, 1979; Fitch and Rosenfeld, 2007).

Thus, neither isochrony nor metrical structure are rigid: both provide a *rhythmic context of expectation* that may be violated for musical effect. Interestingly these two aspects of rhythm seems to trade off with one another, such that highly-syncopated rhythmic styles (West-African, jazz or rock music) tend to have a very steady pulse tempo, while styles with a clear metrical structure often permit more extensive pulse variation and rubato (Temperley, 2004).

Unfortunately, researchers in rhythmic cognition often use the terms discussed above (rhythm, meter, pulse, beat, syncopation) differently, often without definition, and terminology varies among different scholars and eras (Sachs, 1953). In recent empirical work discussing pulse-finding, for example, we see many different terms referring to essentially the same thing. For example, what (Patel, 2006) calls “beat perception” is termed by others beat induction (Honing, 2012), clock induction (Povel and Essens, 1985), or pulse perception (Merker et al., 2009). “Meter” is also extremely polysemous and is sometimes used simply as a synonym for rhythm (e.g., Grahn, 2012), but I will employ the term in a more specific way than this. Given this dangerous lack of shared rhythmic terminology, I define my terminology explicitly below. The goal is to answer “what structure must a listener (implicitly) extract from a rhythmic signal, in order to dance to it appropriately?,” and my answer will be “metrical structure” and *not* simply “pulse.”

### Musical rhythms are trees in time

The central claim of this section is, following (Longuet-Higgins, 1979), that **rhythms are trees in time** or, less concisely, that rhythms represent headed hierarchical groupings of sequential events. My argument will be that for cognitively appropriate movement (e.g., dance) to occur to a rhythm, a metrical tree must be assigned by the listener. Thus, my goal here is to define a kind of “motor schema” that I propose must be inferred by any listener who is able to dance (or clap or tap their foot) to the rhythm.

To make this claim explicit, we must first define “trees” (the type of structure that defines hierarchical systems), and then show how rhythms are a sub-type of a particular type of tree structure.

(1) A *tree* is defined as an acyclic, connected graph.

By this definition, a tree is a type of graph (a mathematical structure composed of *nodes* and *edges* connecting nodes). “Connected” indicates that all of the nodes are connected into one structure (no “floating nodes”), while “acyclic” means that there are no circles or loops in this structure. *Terminal nodes* are those nodes that are connected to only one other (they have “one end free”).

(2) A *rooted tree* is a tree with a single designated *root node*.

The notion of a *root node* is also intuitive: there is some single node from which the entire rest of the tree can be said to emanate, meaning that this node is the parent of all others. Un-rooted trees are also possible (though uncommon in cognitive science).

The central virtue of trees is that any complex rooted tree can be broken down into sub-trees (Martin, 1972). When considered as an independent unit, each subtree also conforms to the definition above, and is thus, in itself a tree with its own root (which constitutes a sub-root in the larger tree). This natural divisibility of rooted trees into subtrees provides an important reason that trees form a natural conceptual structure for considering any system which is made up of parts which form groups that then combine to form larger groups. It also explains why tree structures are so common in computer science and algorithmic design (Skiena, 1998): tree-based problems can easily broken down into simpler and simpler subproblems.

(3) A *headed tree* is a rooted tree whose root is preferentially attached to a one specific terminal, termed the *head*, which plays a special, dominating role in the overall tree.

The notion of roots and sub-roots connects to a central aspect of rhythmic and linguistic trees, namely the notion of “heads” in a tree. The idea is that when several objects combine (be they syllables, notes, words, or phrases), one of them is “singled out” to play a defining role for the larger group. Thus, a noun phrase like “very big brown rat” inherits its category from the head noun “rat”. This large noun phrase can thus, play the same role in a larger sentence as the word “rat” alone, or the smaller phrase “big rat.” Musical structures are also often headed: they inherit their properties from particular components. For example in a chord, a single note is termed (confusingly) the “root” of the chord. This head element provides the name to the chord and defines the relative roles of other notes (which influence, for example, whether the chord overall is major or minor, and thus, sounds relatively cheerful or sombre).

(4) *Hierarchy*: When any of the above tree structures can be assigned to a graph, with complete coverage of both edges and nodes, we say that the graph is *hierarchically structured*.

Note that there is nothing in this definition demanding that a *single* tree form can be assigned to the graph: ambiguity is quite common in both language and music, where multiple possible trees can be correctly assigned to the same data. In such cases the graph structure is ambiguous, and can receive multiple plausible hierarchical interpretations.

### Metrical trees are *headed* trees in time

**Figure 2:**
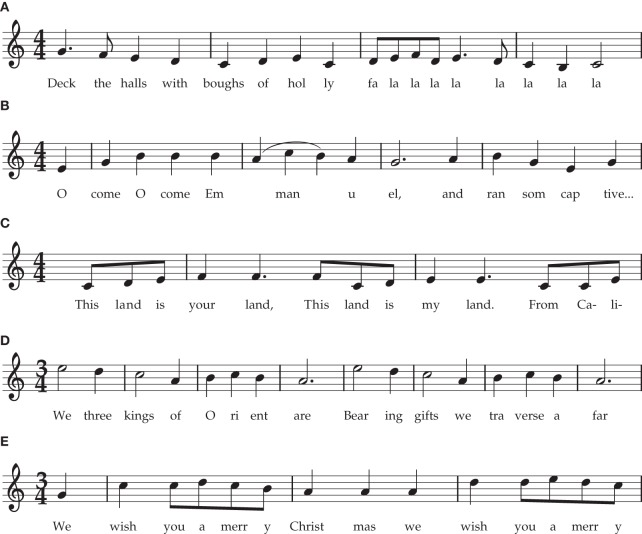
**Familiar Musical Examples of Meter with or without Anacrusis. (A)** March Time (4:4), no pickup: “Deck the Halls.” **(B)** March Time (4:4) with one pickup note: “Oh Come Oh Come Emmanuel.”**(C)** March Time (4:4), three pickup notes: “This Land is Your Land.”**(D)** Waltz Time (3:4), no pickup: “We Three Kings.”**(E)** Waltz Time (3:4), one pickup note: “We Wish You a Merry Christmas.”

My core claim in this section is that **metrical trees are *headed* hierarchies**. In metrical trees, I suggest, the role of the head of a phrase is played by its main downbeat. This is the event on which, when counting out the meter, the word “one” is spoken. This is considered to be a “strong” event, around which other rhythmic events play a subsidiary role, and it is where dancing would commence (or restart after pausing). The head of a rhythmic phrase is not necessarily the first note in a piece of music: it is not uncommon to have pick-up notes leading into a phrase before the first downbeat is played (e.g., in Figure 2C “This Land is Your Land,” in which the downbeat and rhythmic root occurs on the word “your”). Nonetheless, once the music is in full swing, the rhythmic root is always the first beat of each measure, which leads to following very simple definition of the rhythmic head:

(5) The *rhythmic head* of a metrical phrase in music is the first downbeat in the sub-tree encompassing that phrase.

This downbeat or head has a privileged direct relationship to the root node of the phrase. As in linguistics, where the head of a phrase determines the roles of other members of the phrase, in rhythmic trees this initial downbeat provides the organizational structure for the rest of the phrase. Note that “downbeat” in the definition above does *not* necessarily mean a sounded note because, in syncopated rhythms, the downbeat may be silent (a rest), but still play the role of rhythmic head.

One might protest that this claim reflects nothing more than the conventions of Western musical notation. That is, we learn to write music such that the perceived downbeat always comes first in a measure, and we treat all notes leading up to this as an introductory preamble (termed anacrusis or “pickup notes”). If so, the definition would be true only by stipulation. However, I do not think this is the case, because even musically-untrained listeners with no knowledge of notation will start dancing on the downbeat, and if they have a leading leg (right or left), this leg and not the other will come to earth on this downbeat. Furthermore, musically untrained listeners can easily learn to count out a meter, and without specific training will put the “one” on this downbeat. Thus, I argue that headedness is a natural, pre-theoretic aspect of metrical structure, reflected in Western notation but not simply a by-product of it.

If we accept for now the definition of rhythmic head given above, one of the prime virtues of rhythmic hierarchy, differentiating it from most forms of linguistic hierarchy, is the utter simplicity of this definition: once the pulse and meter are known, the head is *always* the first beat in a phrase (measure). In contrast, phrases in language can be head-initial, head-final, or head-medial, and a preference for these different forms varies from language to language, or even within languages (so English noun phrases tend to be head-final (as in “big brown **rat**”) while verb phrases are head-initial (“**went** to the store yesterday”)). Similarly, in musical harmony, the root *may* be the lowest note in a chord, but is not always (e.g., in chord inversions). In contrast, rhythmic phrases are always head-initial in Western music, many New World styles whose rhythms are derived from Africa (jazz, rock, calypso, reggae, salsa…), and at least some West African music (Temperley, 2000, 2001), but see (Arom, 1991). Possible exceptions to this rule include gamelan and Balkan irregular rhythms. This “head initial” characteristic is an important way in which rhythmic hierarchy follows rules that are simpler and more consistent than in many other types of human hierarchical cognition, including phonology or musical harmony.

Importantly, the terminology introduced above does *not* reflect a consensus among rhythm cognition researchers. Often, the terms “pulse” and “beat,” or “rhythm” and “meter,” are treated as synonyms. While some theorists emphasize that metrical structure involves trees (Longuet-Higgins, 1979; Honing, 2012), others specifically deny the notion that metrical cognition involves *headed* hierarchical trees (e.g., Lerdahl and Jackendoff, 1983; Lerdahl, 2013). The latter argue that metrical streams, while possessing an alternating strong/weak patterning, are grouped but *not* arranged into headed hierarchical trees. One reason for this assertion is that the tree structure imposed on a melody (“grouping structure”) is often not aligned with the metrical grid (anacrusis, as in Figures 2B,C,E).

**Figure 3:**
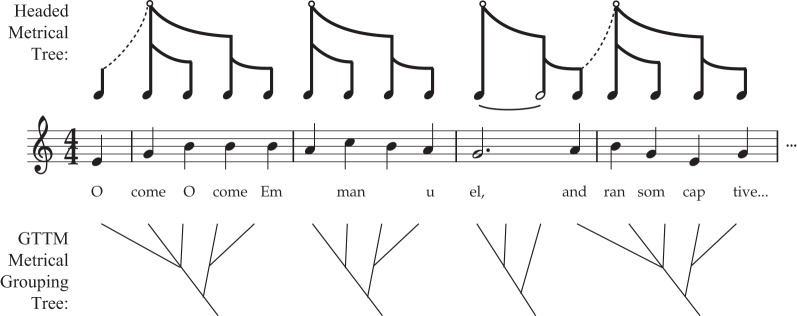
**Comparison of Headed Metrical Trees with GTTM Metrical Grouping Trees.** This figure illustrates two different approaches to anacrusis, comparing the current proposed approach with the (non-headed) GTTM approach of Lerdahl and Jackendoff (1983). In my proposal, the pickup notes *melodically* connected to the root of the following metrical tree (symbolized by the dashed line) but are not part of that tree itself. In the GTTM approach, the anacrusis is part of the tree, and “belongs to” (is subordinate to) the following downbeat.

However, there is no fundamental contradiction in these two approaches (see Figure 3). Anacrusis is explained in the current framework by positing two different trees, melodic and metrical, which are out of phase with one another, somewhat akin to syncopation. The pickup notes can play a useful role for dancers (particularly in partner dancing where two people must be tightly synchronized), by providing a non-danced melodic preamble to clearly indicate when the initial movements should begin. But if a dancer's movements started with the first note of the anacrusis, aligning with the MELODIC grouping rather than with the metrical head (downbeat), the dancer would be making a serious error (and would probably step on their partner's toes).

The model of meter proposed here also differs from that of Cooper and Meyer (1960), who argue that metrical structure propagates higher up in the overall structure of the piece, so that there is a strong/weak distinction even at very long time spans of phrases, motifs or movements (Cooper and Meyer, 1960). While I recognize that such “hypermetricality” can exist [so that for example the chords of the first measure of a piece are more important that those in the second or third bars (Temperley, 2004)], I treat these as an aspect of harmonic structure rather than of metrical structure *per se*, and do not believe that hypermetrical structure is comparable to bar-level accentual pattern. Metrical structures as conceived here are local, purely rhythmic, and confined to the bar level.

To recap, I have three reasons for emphasizing the distinction between pulse and meter. The first is that they can occur independently (e.g., in speech vs. music), and the second is that the computational processes underlying them appear to be quite different. The third and most relevant biologically is that, while we know that many animals are capable of pulse extraction and entrainment (including relatively unsophisticated species like crickets and frogs), we remain in the dark about animal meter perception (see below).

### Levels of rhythmic interpretation: hierarchy vs. recursion

Given these definitions, we can now explore the nature of rhythmic trees in more detail. The first thing to note is that the pulse (tactus) defines an intermediate level of structure, in that pulses can be both combined into larger units (measures, phrases, and above) and subdivided into smaller units [into eighths (quavers), sixteenths (semiquavers), and below]. Thus, the pulse occupies a moderate frequency, on the order of one or two pulses/second, at which listeners can easily dance or tap their feet: this is termed the **tempo** of the musical stream, and hierarchical structure exists both above and below this reference.

The definitions of trees above are very general, and make no assumptions about symmetry or regularity of trees. However, two types of regularity are very common, and important, in rhythmic tree structures. The first regularity is repetitive structure *across* time: that a particular form of grouping repeats itself multiple times across a stream of events (e.g., into recurring groups of three in waltz time, or groups of four in 4:4 or “common” time). Often, an entire piece of music may have the same cross-temporal structure throughout (e.g., most contemporary dance music), though it is not uncommon in Western art music to change meter during a piece. Musical meter is thus, closely connected to the many different accentual syllabic systems used in poetry (Hollander, 2000) such as iambic pentameter (meaning “five groups of syllable-pairs with accents on the second syllable”) or anapestic tetrameter (four groups of syllable-triads, with accents on the first syllable). However, importantly, metrical structure in speech including poetry does *not* typically have the isochrony typical of music: despite early intuitions (Pike, 1945), stressed syllables do not actually occur isochronously in speech (Ramus et al., 1999).

**Figure 4:**
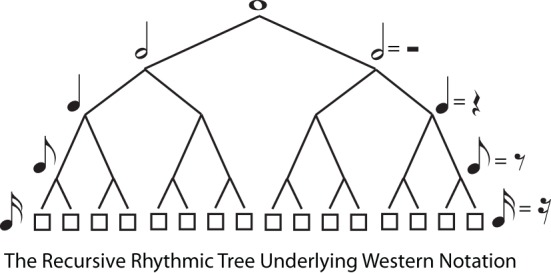
**The Recursive (Branch Symmetrical) Tree Underlying Western Musical Notation.** The tree diagram illustrates that successive binary divisions underlie the division of whole notes into half notes, quarter notes, etc. in Western musical notation. The tree is “recursive” because the same branching structure (binary, in this case) applies at every level of the tree. However, recursive subdivision is *not* always the case in metrical structure, because a meter may be hierarchical (e.g., waltz time in 3:4 time signature) without being recursive.

The second type of hierarchical regularity concerns branching *down* the tree, from root to terminals. Again, the definition of trees imposes no restrictions on this, but a particularly simple form of branching regularity is **recursive structure**, where the same branching pattern continues indefinitely as the tree divides into subtrees. Thus, a binary tree is recursive, because every node that branches at all has precisely two daughter nodes, leading to a highly symmetrical tree form. Recursion is appealing, and powerful, in that the same simple rule can be used to generate trees of unbounded complexity. For example, Western musical notation is based on a recursive, binary branching pattern (whole notes → half notes → quarter notes → etc … or in Britain semibreves → minims → crotchets → …, cf. Figure 4).

Despite the appeal and power of recursion, it is clear that rhythmic trees, while always hierarchical, are not necessarily recursive. Several examples of non-recursive rhythmic trees are illustrated, using familiar tunes as examples, in Figure 2. For instance, in the waltz time “We Wish You a Merry Christmas,” measures are made up of three beats, but these beats would themselves be divided into multiples of two [eighth (quaver) or sixteenth (semiquaver) notes]. Similarly in 6:8 time, an initial binary branch bears three subsidiary branches, and each of these are divided into multiples of two. More unusual, but still attested, are time signatures such as 5:4 (Brubeck's, 1959 “Take Five”), in which each measure contains first a group of three, and then of two, beats. Even in common time 4:4 music, “triplets” are common, which divide each beat into three sub-beats. Thus, recursion is not a defining feature of rhythmic trees, despite the fact that a considerable proportion of Western music is essentially recursive (with pervasive binary branching).

### Sub- and supra-beat interpretation and linguistic stress patterns

Metrical structure provides an interesting parallel between rhythmic and linguistic trees at the supra-pulse (or supra-syllabic) level. As noted, a single pulse can be subdivided into an arbitrary number of subsidiary events: we might call this sub-pulse structure. These can be so rapid as to not even be interpreted as separate notes (e.g., ornaments such as trills, vibrato, or slides), or they can be slow enough as to constitute clearly audible rhythmic substructure [the smallest temporal atom is sometimes termed the “tatum” to distinguish it from “tactus” (Bilmes, 1993; Klapuri et al., 2006)].

In music with English lyrics, downbeats (accented pulses) roughly correspond to the rate at which the stressed syllables of the lyric occur, creating a parallel between word-level phonology and beat-level rhythmic structure. Small integer numbers of beats are also combined into larger units called measures or bars, which are then further combined into arbitrarily large units making up phrases, parts (e.g., verse and chorus), movements, etc. This “bar level” structure has properties more reminiscent of phrasal phonology and syntax: where perceptually complex groups are combined into larger and larger groupings.

Bar level structure in rhythm provides one of the most striking correspondences between music and spoken language: the existence of **metrical accent structure** in both domains. As has often been noted, spoken language and music are very similar, if not identical, in this respect (Liberman, 1975; Idsardi, 1992; Hayes, 1995; Jackendoff and Lerdahl, 2006; Fabb and Halle, 2012). In both cases, there is an alternation of “strong” and “weak” beats over syllables or notes that follows a particular pattern, and these patterns are broadly conserved over many of the world's languages. Stress is an important component of speech prosody, is highly productive phonologically, and appears to play an important role in both speech perception and word learning (Cutler and Norris, 1988; Cutler, 2012). Although the role of stress varies from language to language, it does so in structured ways that lead to well-defined formal universals of stress patterns and stress perception (Hayes, 1995; Cutler, 2012).

The overall notion of stress is intuitive to speakers of languages where stress plays a salient role (such as English, German or Dutch) but less obvious for those whose mother tongues place little emphasis on stress (e.g., French). Nonetheless, early phoneticians found it difficult to find unambiguous markers in the physical signal that indicate strong vs. weak stress: the intuitive idea that stressed syllables are louder turns out to fail, and we now know that various acoustic cues such as length, pitch and vowel timbre all contribute to the percept of stress. This directly parallels the situation in music: stressed syllables are not always louder, and the same multiple cues including pitch, duration, and timbre play a role in determining the “relative prominence” of stressed vs. unstressed notes.

The similarity of cross-linguistic stress patterns to rhythmic metrical patterns is most easily seen in music with lyrics, where the stress pattern of the lyrics must be roughly lined up with that of the musical rhythm; it is also evident in poetry in which stress patterns can play a primary role in defining the overall structure of the verse (Fabb and Halle, 2012). Phonologists debate whether these patterns are only similar, or actually identical (Vaux and Myler, 2012)—a question that must ultimately be decided empirically. But these parallels show that there are deep similarities in the way musical and speech stimuli are arranged, suggesting that rhythmic hierarchy in music and language are not simply abstractions imposed by theorists, but play important cognitive roles in production and perception in both domains.

The crucial difference in musical and linguistic meter is that the pattern of stresses perceived in music map regularly on to the rhythmic tree. Dominating nodes by default receive stronger stress than their subordinates and, as already noted, dominating nodes (heads of subtrees) always come first. This implies a simple and regular pattern emanating from the bar-level head (the “one” of a measure) right down to the smallest subdivisions of the beat: the alternation of strong-weak events propagates all the way down the tree. This clear regularity is refreshingly simple compared to the complexity of language: even in metrical poetry there is no strong head-initial tendency, and iambic meter (stress-second) is as likely as trochaic (stress-first). Thus, I suggest, musical meter has a simpler and more formulaic structure than speech meter (although syncopation provides a means of defying or playing with this underlying metrical simplicity).

### Summary: rhythmic syntax

Adopting these simple and intuitive notion of hierarchy and tree structure, rhythms constitute trees in time. Just like linguistic trees, or other type of musical hierarchy (in the tonal/harmonic domain), rhythmic trees have a head (the downbeat). This head has a privileged projection to the root of the rhythmic tree. As a result, it plays a central role in interpretation of a rhythm: only given the context of the downbeat can the rest of the rhythm be properly interpreted. Downbeat assignment allows the overall rhythmic tree to be inferred, directly effecting the accent structure of all the remaining events (whether notes or rests) that make up the rhythm. Intriguingly, the rules for allocating such stress patterns follow very similar, or identical, rules in rhythm and spoken language.

These considerations lead to three main conclusions. First, because rhythms are trees in time, there are undeniable isomorphisms between rhythmic structures and the phonological/syntactic structures of language. These similarities may result from more general and abstract principles of hierarchical temporal cognition that apply across domains. Second, I suggest that rhythmic structures reduce the complexity of hierarchical structures down to a surprisingly simple and dependable type of hierarchy (e.g., relative to those that complicate syntax). Third, this supports the contention that, when discussing “musical syntax,” we should not think solely of harmonic syntax, but should incorporate rhythmic syntax as another important form. Indeed, because of its simplicity, rhythmic syntax may provide an excellent domain for both neuroscientific and comparative investigations of hierarchically-organized cognition.

## What components of rhythmic cognition are shared with other animals?

It has long been clear that rhythm, and rhythmic synchronization of human pairs or groups, can play an important role in human society and social bonding (McNeill, 1995; Haidt et al., 2008). Recently, a number of studies have examined this phenomenon experimentally, showing that both adults and children show increased pro-sociality after engaging in joint rhythmic activities (e.g., Kirschner and Tomasello, 2009; Wiltermuth and Heath, 2009). Dunbar and his colleagues have suggested that this results from a release of endorphins caused by joint musical or rhythmic activity (Cohen et al., 2010; Dunbar et al., 2012). While there is little consensus at present about the evolutionary origins of these phenomena (Brown, 2000; Merker, 2000; Cross, 2003; Hagen and Bryant, 2003; Fitch, 2012), there can be little doubt that joint rhythmic activity, and synchronization in particular, can have a powerful, biologically-based effect on human emotions. Surprisingly, there has until recently been little exploration of such phenomena in non-human animals (“animals” hereafter). I now turn to a review of this recent and rapidly-growing comparative literature examining animal rhythmic capacities.

There is a long and mostly unwritten tradition considering rhythm perception, and particularly the ability to synchronize to a steady beat, as an unusual ability differentiating humans from other primates (cf. Williams, 1967; Merker, 1999, 2000). Nonetheless there is abundant evidence for such synchronization in a variety of insect and frog species (for audio signaling) and fireflies (for visual signaling), reviewed in Buck (1988); Greenfield (1994). These undoubted examples of pulse extraction and entrainment provide the first, and long known, evidence of a form of animal “rhythm.” These examples illustrate that, given sustained and strong selection, even organisms with small brains can extract a pulse and synchronize to it, at least in a particular input and output modality. This make the rarity of synchronization in general, and in birds and mammals in particular, somewhat enigmatic. However, it is important to note that the more general and cross-modal capacity humans possess to synchronize to essentially arbitrary stimuli (both visual and auditory) at a wide range of tempos. Humans also entrain in multiple motor modalities, including for example body or limb motions, vocalization and even breathing and heart rate (Müller and Lindenberger, 2011). Such flexibility remains undocumented in insects or frogs, where only specific sensory inputs at restricted tempos elicit motor outputs in a single modality (Greenfield, 1994).

There is one suggestion of vocal synchronization in primates: (de Waal, 1988) reports that captive bonobos engage in synchronized “staccato hooting.” Unfortunately no detailed analysis of this phenomenon is available in the published literature, and any latent ability of bonobos to engage in spontaneous synchronization remains poorly documented. Two recent studies with rhesus macaques highlight both similarities and differences between human and primate rhythmic abilities (Zarco et al., 2009; Merchant et al., 2011). In both studies, macaques were trained to tap a key at a regular pulse, and their behavior was compared to human participants. Despite multiple similarities (e.g., a tendency for error to increase as tempo decreased), and a clear and well-developed capacity in both species to compute and remember time intervals (Merchant et al., 2011) there were several key differences. First, humans show a distinct advantage when cued acoustically rather than visually (cf. Patel et al., 2005); this modality difference was not seen in macaques (Zarco et al., 2009). Second, monkeys were unable to synchronize to a metronomic pulse, or continue tapping regularly once such a pulse was removed. Thus, these recent experiments were consistent with the long-held belief in human rhythmic uniqueness.

**Figure 5:**
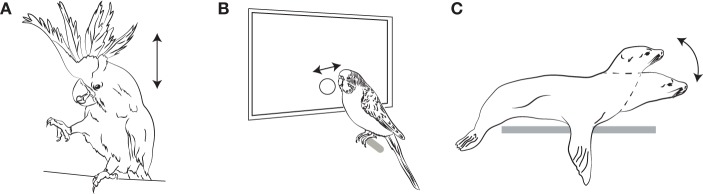
**Figures illustrating Pulse Perception and Synchronization as Recently Demonstrated in Three Non-human Species. (A)** Sulfur-Crested Cockatoo (*Cacatua galerita*); **(B)** Budgerigar (*Melopsittacus undulatus*); **(C)** California Sea Lion (*Zalpohus californianus*). Arrows indicate the type of periodic movements that are entrained to the pulse of the auditory stimulus (head bobs, button taps with the beak, and head bobs, respectively).

Thus, until a few years ago, it was a common assumption in the music cognition community that human rhythmic abilities were unique among birds and mammals, and more general than those in any non-human species. However, this assumption has now been conclusively rejected by a number of recent studies (illustrated in Figure 5).

### Multiple animal species show beat perception and synchronization

The first indications of well-developed synchronization to a musical rhythm in birds came from an unusual source: YouTube (www.youtube.com) videos purporting to show “dancing” in a captive sulphur-crested cockatoo, *Cacatua galerita*, named “Snowball.” This bird was anonymously donated to a bird rescue shelter in Indiana, along with a CD and a note indicating that he enjoyed these songs. When the music was played, the bird began to rhythmically bob his head and lift his legs in time to the music. This was videotaped and uploaded to YouTube, whereby it came to the attention of scientists, many of whom (including myself) were initially sceptical about its veracity. There are many videos on the internet supposedly showing dancing or talking animals which are clearly doctored to synchronize the audio track to the animals' movements, so some scepticism is clearly warranted. But the videos were suggestive enough for Aniruddh Patel and his colleagues to journey to Indiana to probe Snowball's synchronization abilities experimentally.

The results provided the first convincing evidence that an animal can extract the rhythmic pulse from human music and synchronize its movements to that pulse. Patel dubbed this ability (Patel et al., 2009a) “Beat Perception and Synchronization” (BPS) but in terms of the framework adopted above, this ability is better termed “Pulse Perception and Synchronization” or more generally “Pulse Perception and Entrainment” (PPE), since we do not know from these experiments whether metrical structure was inferred. The crucial experiment involved slowing down and speeding up Snowball's preferred song (“Everybody,” by the Backstreet Boys) without changing its pitch, and videotaping Snowball's movements. Although Snowball did not always synchronize to the beat (he seems to require some time to “get into it”), once a synchronized state is reached, he bobbed his head in nearly perfect time to the music. In particular the average phase relation between head bobs and pulses is not significantly different from 0°, indicating that he bobbed neither before nor after the downbeat, but directly on it. This would be very difficult in a purely reactive situation (where a listener moves only after hearing the relevant event), but instead indicates a predictive situation where a pulse is inferred, and movements synchronized to it.

The discovery of PPE in Snowball immediately raised many questions about the origins and frequency of this ability in other cockatoos, or other species. In an innovative approach to this question, Adena Schachner and colleagues performed a large-scale analysis of YouTube videos purporting to show “dancing animals” (Schachner et al., 2009). Schachner and colleagues sifted through more than 1000 such videos, testing whether the animal subjects maintained a consistent phase relative to the downbeat and/or matched its tempo. Many of the videos either showed no evidence for either of these criteria, or showed obvious evidence of *post-hoc* doctoring. However, 33 videos revealing what appeared to be PPE were uncovered. Among the 15 species in which solid evidence for PPE was observed, an astonishing 14 were parrots (order Psittaciformes); the only exception was a single Asian elephant. Schachner and colleagues also presented experimental analyses of both Snowball and another parrot (the famed African gray parrot “Alex”), both consistent with the conclusions of Patel and colleagues. Despite hundreds of videos showing dancing dogs (an entire competitive domain of dog training termed “canine freestyle” exists in which dogs supposedly dance to music), none of these showed convincing evidence of PPE.

These findings have galvanized the field of animal rhythm research, and have led to a number of more controlled laboratory studies. The abilities of parrots to synchronize have been further studied by Hasegawa et al. (2011) in eight budgerigars (*Melopsittacus undulatus*), who were readily trained to tap to an acoustically- and visually-indicated tempo at a wide range of frequencies. While budgies learned the task more easily for slow tempos (1200–1800 ms period), the budgies tapped more accurately to more rapid tempos (450–600 ms). A phase analysis showed that all of the budgies tended to lead the beat slightly once the task was acquired (a phenomenon frequently seen in humans), again indicating that a merely reactive process is very unlikely to account for the details of their timing. To date, this paper remains the only laboratory demonstration of PPE in multiple individuals of the same species, and the ease with which budgies can be kept in the lab suggests they make an excellent model species to study these phenomena further.

An even more impressive demonstration of mammalian PPE comes from a recent paper by Cook et al. (2013) showing excellent synchronization of head bobs in a California sea lion, *Zalophus californianus*, named Ronan. This study is exemplary from a methodological viewpoint, and Cook and colleagues took particular pains to avoid potential confounds like unconscious cueing by human experimenters. Crucially, after she was trained to synchronize to a simple repetitive sound, at two different tempos (80 and 120 BPM), Ronan spontaneously generalized to five new tempos. Equally important, after training solely with a simple metronomic stimulus, she generalized spontaneously to complex recorded music at various tempos. This surprisingly suggests that, once the motor task of periodic motion synched to a sound was learned, the perceptual task of extracting the beat from a complex acoustic signal was comparatively simple for this sea lion. An important control experiment incorporated “missing” beats, omitted from an otherwise regular tempo. Ronan did not omit her head bobs preceded by such missing beats, demonstrating that she did not simply react to auditory events (with her reactions happening to coincide with later events), but rather extracted the pulse tempo and used it to entrain her own inner pulse.

A final recent study in this vein demonstrated spontaneous synchronization to a repeated keyboard note by one common chimpanzee *Pan troglodytes* (Hattori et al., 2013). A group of three chimpanzees were trained to tap on alternating, briefly illuminated keys of a MIDI keyboard. They were required to learn to tap alternating keys a minimum of thirty consecutive times for a food reward (“training”), and after consistently meeting this criterion moved on to a test stage in which a repeated “distractor” note (different from the one produced by their tapping) was played at a consistent tempo (400, 500, or 600 ms inter-onset interval). Reward was given for completing 30 taps, regardless of any synchronization or lack thereof. Nonetheless, one of the three chimpanzees, an elderly female named “Ai,” spontaneously aligned her taps (mean of roughly 0° phase) to this steady auditory pulse. Ai did not show synchronization to the other two tempos, and the authors hypothesized that her successful synchronization to the 600 ms tempo stemmed from the fact that her spontaneous tapping frequency (about 580 ms) was very close to this. The two other chimpanzees showed no evidence of synchronization. Although the restriction to one of three animals and a single tempo suggests that chimpanzee's synchronization abilities are quite limited compared to those of parrots or sea lions, this is an important finding, and essentially the first well-controlled study demonstrating some (but not all) components of PPE in any non-human primate.

### Interpreting these recent animal PPE findings

In summary, it is now clear that many animals can entrain to species-specific signals, and several different animal species, most prominently multiple parrot species and a California sea lion, are able to extract a pulse from music and entrain or synchronize their movements to this pulse. The analysis of YouTube videos by Schachner and colleagues strongly suggest that a wide variety of other species with prolonged exposure to human music (e.g., pet dogs and cats, and a wide variety of pet birds) *do not* spontaneously entrain to human music. What are we to make of the pattern of phylogenetic distribution of entrainment in animals?

An influential hypothesis in the study of animal rhythm is due again to Patel, who suggested that the capacity of a species to show entrainment may derive from their capacity for complex vocal learning (Patel, 2006). Several subsequent studies of animal entrainment have explicitly tested this hypothesis (Schachner et al., 2009; Hasegawa et al., 2011; Cook et al., 2013; Hattori et al., 2013). Complex vocal learning (specifically vocal production learning) is an unusual ability in the animal kingdom, but nonetheless has arisen multiple times in both mammalian (Janik and Slater, 1997, 2000) and avian evolution (Nottebohm, 1975; Jarvis, 2004). The significance of vocal learning for the evolution of human speech has long been recognized (Nottebohm, 1975; Fitch, 2000; Egnor and Hauser, 2004), because spoken language requires an open-ended vocabulary that must be learned if it is to be shared by a community.

Patel proposed that selection for vocal learning might lead to a capacity for rhythmic entrainment as a side-effect (Patel, 2006). The basic intuition behind this idea is straightforward: vocal learning requires an unusually close collaboration between auditory and vocal motor systems, presumably underpinned by neural connectivity that may be unusual in the vertebrate brain. Once such connections are in place, driven by selection for vocal learning, they lead to a propensity for auditory input to modulate motor behavior *in general* (not just vocal motor behavior), essentially as an unselected by-product of selection for vocal learning.

The early data showing PPE in parrots was clearly consistent with this hypothesis, since parrots are famed vocal learners (Nottebohm, 1975; Jarvis, 2004; Pepperberg, 2005). The more tenuous observation of apparent entrainment by an Indian elephant in a YouTube video (Schachner et al., 2009) is also consistent, because a capacity for complex vocal learning (speech imitation) has recently been demonstrated in an Indian elephant (Stoeger et al., 2012). Despite these consistent examples, it is important to note the *absence* of evidence for entrainment in a wide variety of vocal-learning species, including particularly songbirds kept in human homes, and vocal learners like dolphins or orcas that in captive settings are often exposed to music. Although it is dangerous to draw strong conclusions from this absence of evidence (perhaps with training dolphins or mynahs can learn to entrain), these examples suggest that vocal learning may be a necessary, but not sufficient, precondition for PPE (Fitch, 2009; Patel et al., 2009b; Schachner, 2010).

However, the more recent data for chimpanzees and sea lions call even this weakened version of the hypothesis into question. The absence of any capacity for complex vocal learning in chimpanzees and other great apes is well documented (Furness, 1916; Yerkes and Yerkes, 1929; Hayes, 1951; Kellogg, 1968), and recent data suggesting a modicum of vocal flexibility in apes (Marshall et al., 1999; Crockford et al., 2004; Reynolds Losin et al., 2008) do not challenge this statement, since the calls modified in these studies are part of the innate species-typical repertoire, and not novel utterances requiring auditory input (cf. Fitch and Zuberbühler, 2013). Despite this, at least one chimpanzee, Ai, was able to synchronize to a pulse, and did so spontaneously without training. This does *not* constitute full PPE since Ai's entrainment did not require extraction of a pulse from a complex musical stimulus, nor did Ai generalize to tempos other than one close to her spontaneous rate of tapping. It remains possible that the rather rough alignment seen between taps and pulses is a chance occurrence due to the two having very close periodicities; more research is necessary to find out. Nonetheless, these results seem to indicate simple entrainment in some individuals of a species whose capacity for vocal learning is very limited, or entirely absent.

More telling evidence against the vocal learning hypothesis is the clear and convincing demonstration of PPE in a California sea lion (Cook et al., 2013). Sea lions are otariids, or “eared seals,” members of one of three families of pinnipeds. There is good evidence for vocal learning from the other two pinniped families (walruses and phocids or “true seals”), particularly for the harbor seal *Phoca vitulina* (Ralls et al., 1985; Hanggi and Schusterman, 1994; Janik and Slater, 1997). Both walruses and phocids “sing” territorial songs during the mating season that are likely to have a learned component (Sjare et al., 2003; Van Parijs, 2003; Schusterman and Reichmuth, 2008). In contrast, there is no evidence for complex vocal learning in otariid seals, and California sea lions specifically, although they are very common in zoos. Although sea lions can easily be conditioned to vocalize on command (Schusterman and Balliet, 1970; Schusterman, 2008), such vocal conditioning is also possible in many other mammals including monkeys and apes (Adret, 1992; Fitch and Zuberbühler, 2013). This lack of evidence for vocal production learning in sea lions stands in sharp contrast to Ronan's clear capacity for entrainment, and her easy generalization to multiple tempos and musical pieces. Thus, the sea lion data argue against the idea that entrainment is a by-product of vocal learning, or that complex vocal learning is a necessary precondition of PPE.

In summary, this series of studies, appearing within the last five years, has catapulted the study of animal rhythmic abilities into a new era, sweeping aside a long-standing presumption that complex PPE is uniquely human. Although sample sizes remain small, even a single instance of PPE in an animal is enough to render a “uniquely human” claim false, and it is now clear that, in the case of both pinnipeds and parrots, animal rhythmic abilities can be brought into the laboratory and analyzed in controlled experiments (Hasegawa et al., 2011; Cook et al., 2013). The rise and (perhaps) fall of Patel's vocal learning hypothesis illustrates the importance of clear, testable hypotheses in this endeavor, since much of this recent work has been galvanized by Patel's suggestion.

Despite this excitement and promise, it is clear that these are early days, with more work on a much wider variety of species required. Importantly, as emphasized above, synchronization and entrainment to a pulse is only one component of musical rhythm: the other is hierarchical metrical structure. It remains unclear whether the animals discussed above simply perceive a periodic pulse, or construct a more complex metrical representation. Here the comparative data is sparse, but there are some recent promising developments that provide preliminary indications.

### Do animals perceive meter?

While it is relatively straightforward to determine that an organism perceives and entrains to a periodic pulse, it is much more difficult to determine from behavioral data whether a listener assigns relative prominence to periodic events or constructs from them a hierarchical metrical structure. Impressionistically, both Snowball the cockatoo and Ronan the sea lion appear to align their head bobs “properly” on downbeats, but further experiments would be necessary to determine if they start on the “one.” Similarly, experiments show that both tamarin monkeys and rats can distinguish between languages with different rhythmic classes better than those in the same rhythmic class (Toro et al., 2003; Tincoff et al., 2005), but such successful discriminations could be based on a multitude of acoustic cues other than metrical grouping.

A promising perspective on this issue is provided by non-invasive recording techniques like evoked responses in the electro-encephalographic (EEG) signal. Henkjan Honing and colleagues have recently demonstrated this potential, using EEG as a signal of metrical perception in adult humans (Ladinig et al., 2009) and infants (Winkler et al., 2009). These experiments make use of the phenomenon of the mismatch negativity (MMN) in EEG signals: when a regular and predictable auditory sequence is established, deviations from the regular pattern (“oddballs”) evoke a reliable negative-going response. Crucially, this response is elicited even when predicted events *do not* occur. Thus, if a predicted event is left out, this absence nonetheless triggers a synchronized MMN.

In an innovative experimental design exploiting these facts, Honing and colleagues constructed musical rhythms that were either consistent with or violated a simple 4:4 metrical structure, played using standard drum sounds (Ladinig et al., 2009; Honing, 2012). A control sound involves all metrical slots being filled (e.g., a rapid high-hat cymbal sound playing on all sixteenth notes, and with bass and snare drum sounds on the downbeats). Experimental sound sequences involve two kinds of omission. When the sound that would occur on an unaccented slot is omitted, this is consistent with metrical expectation. In contrast, if unaccented events occur but the *downbeat* is left out, this constitutes a strong violation (syncopation). Consistent with this prediction, both adult and infant human listeners show a strong MMN to a missing downbeat (metrical violation) but a smaller or absent response to metrically-acceptable omissions. Electrodes were placed at the F3, Fz, F4, C3, Cz, and C4 scalp locations, and maximum amplitudes observed at the Cz (central) electrode. This is true even in adult listeners with little musical training, and may occur pre-attentively (Ladinig et al., 2009, 2011). These studies provide empirical evidence for ideas that theorists have long taken for granted, and demonstrate the potential for EEG to be used as an assay for metrical cognition (but for cautionary notes see Honing, 2012).

Recent work has applied this same paradigm to two rhesus macaques (Honing et al., 2012). Because use of surface EEG in animals is a relatively recent phenomenon, initial experiments simply demonstrated that a MMN can be observed in macaques, first using a tonal “oddball” task (pitch deviant) and then an omission deviant, where an expected tone was replaced by silence. Five electrodes were used (Fz, Cz, Pz, F3, F4). In both cases a frontal/central MMN was observed, though it was relatively small in the omission case, and varied in sign (negative in one monkey and positive in another). In the crucial third experiment, monkeys heard the same type of drum patterns used in humans, and were exposed to a similar range of stimuli. Here, again, omissions elicited significant mismatch potentials. Crucially, however, those did not differ significantly for metrically-consistent and inconsistent omissions. This suggests that although the monkeys (1) were able to extract temporal regularity from an auditory signal and note an omission within it (basic regularity detection) and (2) recognized some grouping in the repetitive drum pattern (enough to notice deviations from it), they did *not* assign a hierarchical metrical structure to this pattern in which the downbeat head of the rhythmic phrase was differentiated from other non-root events. Thus, at least in these two monkeys, it seems that no hierarchical structure was assigned to the acoustic stream of events. Similar results have been found in pigeons, with timing abilities evident but no evidence for pulse extraction or metrical grouping (Hagmann and Cook, 2010). These studies clearly illustrate the potential for timing perception to occur in the absence of human-like rhythmic cognition with pulse and metrical structure.

While again we are forced by these experiments to interpret absence of evidence, the approach taken in these studies is a general one, and could readily be applied to species in which we do have evidence of PPE such as parrots and sea lions. We can only hope that, in the next five years of this fast-moving research area, there will be studies examining the capacity of these species to differentiate metrical structures as well as pulse periodicity. For while the current comparative data enable a confident statement that some animals can extract a pulse and entrain and/or synchronize to it, we as yet have no positive data concerning metrical structure perception by any non-human animal.

## Implications and future research directions

The new data demonstrating entrainment abilities in animals have ushered in a new era in which the biological and neural roots of rhythmic cognition can be studied empirically. This should open the door to comparisons of the large existing database concerning human rhythmic abilities (Fraisse, 1978; Jones and Boltz, 1989; Desain and Windsor, 2000; Drake et al., 2000; Repp, 2005; McAuley et al., 2006; Honing, 2009) with those of other animals. I thus predict that the next decade will bring fundamental new insights into the biological bases of human rhythmic abilities. I end by sketching some lines of research that I suspect may be particularly worthwhile.

First, we now have an opportunity to explore the neural bases of pulse perception in animals. While both EEG and intracranial work in monkeys certainly should continue, it seems likely that the most profound insights will come from entraining species like parrots or sea lions. Budgerigars also provide an excellent potential species for such investigations: they are small, easily kept in the laboratory, and with a long history of both behavioral experimentation (e.g., Okanoya and Dooling, 1987; Dooling and Brown, 1990; Farabaugh et al., 1994) and neuroscientific investigation (e.g., Paton et al., 1981; Manogue and Nottebohm, 1982; Dooling et al., 1987; Hall et al., 1993; Striedter, 1994; Durand et al., 1998). The possibility of non-invasive measurement of ERPs in these and larger parrots should also be explored: this would enable an investigation of metrical structure attribution in these birds along the lines of Honing et al. (2012).

Second, the work of Hattori et al. (2013), along with other recent work on rhythm in bonobos (Edward Large, pers. comm.) suggests that the synchronization abilities of chimpanzees may have been unjustly neglected. Perhaps, given the correct context, great apes can learn to synchronize their actions to an auditory pulse or music. Given that African great apes (chimpanzees, bonobos, and gorillas) are among the only primates that show spontaneous hand-drumming in the wild (Schaller, 1963; Goodall, 1968; Arcadi et al., 2004), both entrainment and metrical abilities in these species deserve more empirical attention (reviewed in Fitch, 2006). Although the entrainment abilities of Ai are rather limited relative to those of humans or some birds, it will be extremely worthwhile to explore these further, both to determine the range of tempos to which chimpanzees can entrain and to determine whether they can entrain to more complex musical rhythms. The latent abilities of chimpanzees who do not spontaneously entrain could be further explored using operant training and positive feedback.

Third and most fundamental, I hope that researchers exploring the rhythmic capacity of animals recognize the central importance of metrical structure to human rhythmic cognition, and develop assays for meter perception in those species known to exhibit PPE. For example, hints of metrical structure in Ai's performances might be gleaned by examining the details of timing or velocity of her key presses. The head bobs of parrots or sea lions could be analyzed for any multi-level patterns that could reflect an internal differentiation of strong and weak beats; if so, how do those relate to the metrical patterns inferred by humans?

Moving to neuroscience, there been a strong bias to study harmony and pitch perception in the music cognitive neuroscience literature, but rhythm has received increasing attention in recent years. This may have practical clinical importance: a recent study suggests that the success of music therapy for aphasia patients hinges more upon rhythm than melody (Stahl et al., 2011). Brain imaging studies quite consistently indicate that human rhythmic abilities involve some privileged interaction between auditory and motor regions of the brain (Grahn, 2012). For example, traditional “motor” regions including both supplementary motor cortex and basal ganglia both appear to play a role in beat *perception* (Grahn and Brett, 2007; Geiser et al., 2008; Grahn, 2009a,b), and humans show superior abilities synchronizing to acoustic than to visual sequences (Patel et al., 2005). Furthermore, simple motor activities like tapping to a beat appear to enhance our auditory time perception abilities (Manning and Schutz, 2013). This suggests that, even if Patel's vocal learning hypothesis does not apply across all animal species, it still provides possible insights into the connection between motor activity, temporal perception, and rhythm in our own species. Unfortunately, since pulse and meter are often not clearly distinguished in the neuroscientific literature, few studies have specifically singled out metrical tree assignment for neuroscientific study (but see Chen et al., 2006, 2008; Iversen et al., 2009).

One point that becomes clear after distinguishing pulse perception from metrical assignment is that the former is music-specific, while the second is largely shared between speech (especially poetry) and music. While there is growing consensus among theorists that this reflects an important area of computational overlap between music and language (Lerdahl and Jackendoff, 1983; Jackendoff and Lerdahl, 2006; Fabb and Halle, 2012; Vaux and Myler, 2012), this hypothesis has received little empirical examination. To what extent is this apparent overlap a result of shared computational machinery, or even dependent upon the same brain areas (Geiser et al., 2008)? As I have suggested above, metrical structure provides a particularly simple and elegant form of hierarchical structure in human cognition, and offers unique opportunities for exploration of more general aspects of hierarchical and syntactic perception.

In addition to the multiple motor areas involved in pulse perception (Grahn, 2012), it will be of particular interest to determine which brain regions play central roles in meter perception, e.g., using the types of stimuli pioneered in (Ladinig et al., 2009). Given the importance generally assigned to Broca's area in the perception and generation of hierarchical structures in language, as documented in much recent brain imaging research (e.g., Friederici, 2002; Hagoort, 2005; Friederici et al., 2006), it would be particular interesting to know what role this region plays in the construction of rhythmic hierarchies. This region, and its right hemisphere homolog, are preferentially activated by tasks involving harmonic syntax (Maess et al., 2001; Koelsch et al., 2002; Levitin and Menon, 2003), so it would not be surprising to find that parts of Broca's area also play a role in constructing hierarchical structures during metrical perception (for intriguing hints see Vuust et al., 2006; Geiser et al., 2008).

Broca's region, and Brodmann's areas 44 and 45 in particular, are among the most greatly expanded cortical areas known in humans: Area 44 is 6.6 times larger in humans than in chimpanzees and Area 45 is 6.0 times larger (Schenker et al., 2010). This suggests a recent expansion of our abilities in whatever computations these regions support, and temporal hierarchy-building is one of the prime candidate operations. The inferior frontal gyrus also exhibits enlarged and modified connectivity to posterior brain regions, including both associative areas and auditory areas, relative to macaques or chimpanzees (Rilling et al., 2008; Friederici, 2009). If indeed these circuits play a key role in human rhythmic cognition, and building metrical structures in particular, these anatomical changes may help to explain why our rhythmic abilities are both preferentially tied to the auditory domain, and greatly expanded relative to those of chimpanzees. Testing this suggestion requires experimental designs that draw a clear distinction between pulse perception and the building of metrical hierarchy.

In summary, this is an exciting time for scientists interested in the biology of rhythm, with multiple new research possibilities ripe for exploration. The long-presumed uniqueness of human rhythmic abilities is no longer tenable, and we can now begin employing the power of the comparative method to the evolution of a central aspect of human music and speech. Nonetheless, much remains unknown, and whether animals capable of entrainment also perceive metrical structure remains an open question. The answer to this question (which I hope is positive for at least some species) is particularly important given the fact that metrical structure provides a clear and well-documented parallel between speech and music. Thus, a finding that some animals perceive metrical structure would be relevant not just for our understanding of the biology and evolution of music, but for language as well. By clarifying the distinction between pulse and meter, I hope that the current paper helps to speed this discovery process along.

### Conflict of interest statement

The author declares that the research was conducted in the absence of any commercial or financial relationships that could be construed as a potential conflict of interest.
